# A Non-Intrusive Pressure Sensor by Detecting Multiple Longitudinal Waves

**DOI:** 10.3390/s16081237

**Published:** 2016-08-05

**Authors:** Hongliang Zhou, Weibin Lin, Xiaocheng Ge, Jian Zhou

**Affiliations:** 1 State Key Laboratory of Industrial Control Technology, College of Control Science and Engineering, Zhejiang University, Hangzhou 310027, China; vlinwb@gmail.com; 2 School of Computing and Engineering, University of Huddersfield, Huddersfield HD1 3DH, UK; x.ge@hud.ac.uk; 3 College of Electrical Engineering, Zhejiang University, Hangzhou 310027, China; zj99@mail.hz.zj.cn

**Keywords:** non-intrusive, pressure sensor, ultrasonic, longitudinal waves

## Abstract

Pressure vessels are widely used in industrial fields, and some of them are safety-critical components in the system—for example, those which contain flammable or explosive material. Therefore, the pressure of these vessels becomes one of the critical measurements for operational management. In the paper, we introduce a new approach to the design of non-intrusive pressure sensors, based on ultrasonic waves. The model of this sensor is built based upon the travel-time change of the critically refracted longitudinal wave (L_CR_ wave) and the reflected longitudinal waves with the pressure. To evaluate the model, experiments are carried out to compare the proposed model with other existing models. The results show that the proposed model can improve the accuracy compared to models based on a single wave.

## 1. Introduction

Pressure vessels are widely used in many fields, such as chemical plants, power stations, etc. In many cases, pressure vessels are safety critical because they contain flammable, explosive, virulent, or corrosive materials. Accidents due to explosion and leakage of the contents will lead to serious consequences, and high pressure is one of the most identified causes leading to these kinds of accidents. Therefore, it becomes critically important to measure the pressure of these vessels in an accurate and convenient manner.

It is very common that the pressure is measured by pressure gauges where it is able to directly touch the materials contained. However, it is not always applicable or cost-effective to mount such pressure gauges. For example, sometimes it will require the upgrade of a lot of old equipment, or the installation of gauges will change the integrity of the vessel, which may lead to other safety issues. There is another approach to measure the pressure—non-invasive approaches [1]. There are several proposed methods of this approach, such as the strain gauge method [2], the capacitor method [3], and the ultrasonic method [4,5,6,7]. These methods can solve some problems, but there is still room for improvement in terms of the accuracy. In [2], Hoffmann discusses that the accuracy of the strain gauge method is heavily affected by the environment—particularly the temperature and humidity. In [3], it was stated that the capacitor method is only applicable to small-diameter pressure vessels, and its accuracy is sensitive to the type of medium inside the pressure vessel and the environment.

The ultrasonic method is more promising and has attracted more interest since it was proposed, because it has been identified that the ultrasound wave can carry much richer pressure-related information. Guers et al. [1,5] established the relationship between the amplitude of an ultrasonic wave propagated inside the vessel and the vessel pressure, and used the reflected ultrasonic signal from the fluid–vessel interface to measure the pressure. However, it is greatly influenced by the type of medium inside the vessel. Zhang et al. [4] found that the travel-time changes of surface waves changed linearly with the pressure, and applied the surface waves to the pressure measurement of thin-walled vessels. However, the propagation of the surface wave is severely affected by the roughness condition of the vessel wall. Ling et al. [6] applied L_CR_ (the Critically Refracted Longitudinal) wave and Rayleigh wave simultaneously to reduce the temperature effect, but the system is complicated because it needs at least four ultrasonic probes. Bi et al. [7] achieved higher sensitivity than the L_CR_ wave and Rayleigh wave by employing the reflected longitudinal waves and temperature compensation. In all these methods above, the difference of waves’ travel-time under different pressure is considered to be small in both the reflected longitudinal waves and the L_CR_ wave, and this limits the accuracy of pressure measurements.

Meanwhile, temperature is another major factor which will affect the ultrasonic properties [8,9,10]. Thus, in order to increase the capability of interference mitigation and to improve the accuracy of measurement, we proposed a non-invasive method of measuring pressure by taking account of L_CR_ and the multiple reflected longitudinal waves. The rest of the paper is structured as follows: in Section 2, we explain the acoustoelastic effect of the ultrasonic wave, its application in pressure measurement, and propose a multi-waves fusion algorithm used in our proposed measurement method; in Section 3, we describe the design of a new pressure sensor based on our method and the prototype measurement system; in Section 4, we discuss our experiment and analyze its results; finally, in Section 5, we summarize the conclusions that can be drawn from this paper.

## 2. Pressure Measurement Method Based on L_CR_ Wave and Reflected Longitudinal Waves

### 2.1. Generation of Multiple Waves in the Vessel Wall

**Figure 1:**
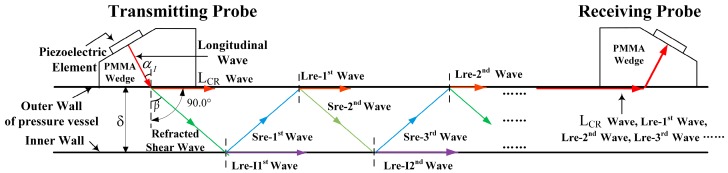
Propagation of multiple waves inside the vessel wall. L_CR_ wave: critically refracted longitudinal wave; Lre-1^st^: first reflected longitudinal wave; Lre-I1^st^: the first inner reflected longitudinal wave; PMMA: polymethyl methacrylate; Sre-1^st^: first reflected shear wave.

**Figure 2:**
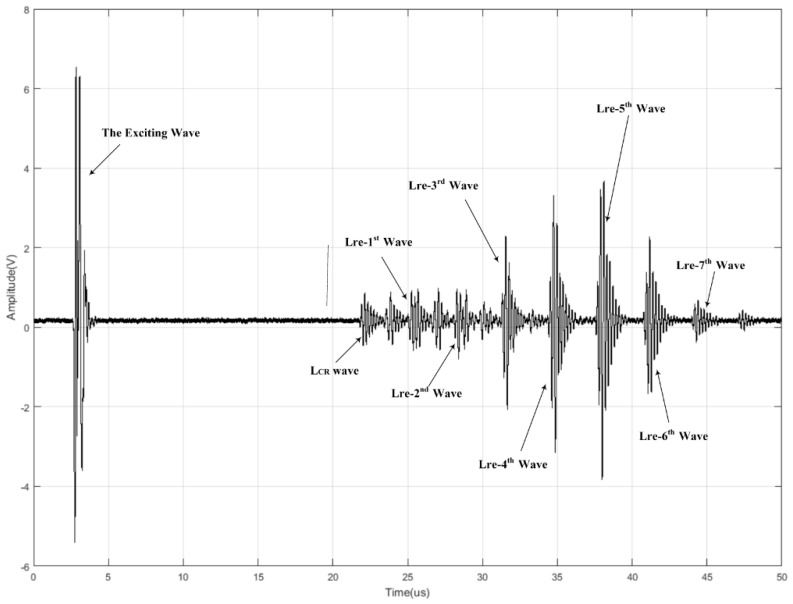
Ultrasonic signal received by the receiving probe.

When a longitudinal wave is generated from a polymethyl methacrylate (PMMA) wedge and penetrates the outer wall of the pressure vessel with the first critical angle ($\alpha_{I}$) (as shown in Figure 1), there will be waveform conversions at the interface. According to Snell’s Law, the origin wave will split into two waves: a L_CR_ wave and a refracted shear wave. The L_CR_ wave propagates along the outer wall and will be received by the receiving probe. The refracted shear wave will reach the inner wall of the pressure vessel with the refracted angle (β), and then the first inner reflected longitudinal wave (Lre-I1st) and the first reflected shear wave (Sre-1st) will be generated. The first reflected shear wave (Sre-1st) will reach the outer wall and generate the first reflected longitudinal wave (Lre-1st) and the second reflected shear wave (Sre-2nd), and so on. As shown in Figure 2, the receiving probe at the other end of vessel will receive multiple waves, including the L_CR_ wave and reflected longitudinal waves such as the Lre-1st wave and the Lre-2nd wave, etc. Among the waves received by the receiving probe, the L_CR_ wave will always arrive first because it travels the shortest distance and it travels with the velocity of a longitudinal wave, which is about twice the velocity of a shear wave. The Lre-1st wave reaches the receiving probe with a time delay Δt (described by Equation 1) after the L_CR_ wave. In this analogy, other adjacent waves will have the same time delay Δt between them. By utilizing this pattern, we can identify these waves at the receiving probe, and we analyze these waves to compute the pressure inside of the vessel.

(1) $$\Delta t=\frac{2\delta }{V_{S}\cos\beta }-\frac{2\delta \tan\beta }{V_{L}}$$

where δ is the thickness of the pressure vessel wall, $V_{S}$ and $V_{L}$ are the velocity of the shear wave and the longitudinal wave, respectively. 

### 2.2. The Acoustoelastic Effect and the Relationship between Pressure and Travel-Time Change 

Hughes and Kelly [11] developed the relationship between the wave speeds and the strain in the pressure vessel, which can be expressed as:

(2a) $$\rho_{0}V_{AA}^{2}=\lambda +2\mu +\left(2l+\lambda \right)\left(\varepsilon_{A}+\varepsilon_{R}+\varepsilon_{C}\right)+\left(4m+4\lambda +10\mu \right)\varepsilon_{A}$$

(2b) $$\rho_{0}V_{AR}^{2}=\mu +\left(m+\lambda \right)\left(\varepsilon_{A}+\varepsilon_{R}+\varepsilon_{C}\right)+4\mu \varepsilon_{A}+2\mu \varepsilon_{A}-\frac{1}{3}n\varepsilon_{C}$$

where $V_{AA}$ and $V_{AR}$ are the longitudinal wave velocity and shear wave velocity along the axial direction of the vessel wall respectively. $\varepsilon_{A}$, $\varepsilon_{R}$, and $\varepsilon_{C}$ are the strains along the axial, radial, and circumferential directions of the vessel wall, respectively. $\rho_{0}$ is the initial density of the pressure vessel. λ and μ are the second-order elastic constants, while *l*, *m*, and *n* are the third-order elastic constants.

By using Hooke’s Law [12], we can model the relationships between the strain components in three orthogonal directions and stress as:

(3a) $$\varepsilon_{A}=\frac{1}{E}\left(\sigma_{A}-\upsilon \sigma_{C}\right)$$

(3b) $$\varepsilon_{R}=-\frac{\upsilon }{E}\left(\sigma_{A}+\sigma_{C}\right)$$

(3c) $$\varepsilon_{C}=\frac{1}{E}\left(\sigma_{C}-\upsilon \sigma_{A}\right)$$

where *E* is the elasticity modulus of vessel material, υ is Poisson’s ratio, and $\sigma_{A}$ and $\sigma_{C}$ are the axial stress and the circumferential stress, respectively.

In the thin-shell theory [13], the stress field in the vessel wall is two-dimensional, including the stress in the axial direction and in the circumferential direction, as described by:

(4a) $$\sigma_{A}=\frac{pR}{2\delta }$$

(4b) $$\sigma_{C}=\frac{pR}{\delta }$$

where *p* is the internal pressure of the vessel, *R* is the average radius of the vessel, and δ is the thickness of the wall.

According to the above analysis, we are aware that the velocity of the longitudinal wave and shear wave along the axial direction are affected by the pressure in the vessel. In reality, the velocity of the ultrasonic wave is high if the vessel is made of steel. The speed of the longitudinal wave is about 5800 m/s, and that of the shear wave is about 3100 m/s [14]. While the velocity change is relatively small, it is reasonable to assume that the velocity change is linear with the change of pressure to some extent.

It is worth mentioning that elastic constants λ, μ, *l*, *m*, *n* and elasticity modulus *E* are all affected by the temperature of the vessel wall. So, the relationship between the wave velocity and the pressure is also affected by the temperature. In practical application, the wave velocity can be obtained by measuring the propagation time between fixed transducers.

### 2.3. The Multi-Waves Fusion Algorithm

The relationships between the pressure and propagation time of different ultrasonic waves have already been studied in our previous works. For example, Rayleigh wave has been discussed in [6], the L_CR_ wave in [6], and the reflected longitudinal wave in [7]. However, the accuracy of pressure measurement depends heavily on the accuracy of the propagation time measurement, which can be affected by multiple factors, including noise, temperature, etc. Furthermore, the change in travel-time induced by pressure is very small in value. So, it is difficult to achieve precise measurement of pressure using a “single” ultrasonic wave method.

Data fusion techniques combine data from multiple sensors and related information from associated data. This can help in improving accuracy and analyzing more specific inferences than in the case of a single sensor alone [15]. As discussed in the previous section, a single receiving probe will at least be able to detect the L_CR_ wave and several reflected longitudinal waves—such as the Lre-1st wave and the Lre-2nd wave. By using data fusion techniques, it is reasonable to believe that pressure measurement accuracy can be improved.

## 3. The Ultrasonic Pressure Sensor and the Experimental System

### 3.1. The Pressure Sensor Based on Ultrasonic Wave

**Figure 3:**
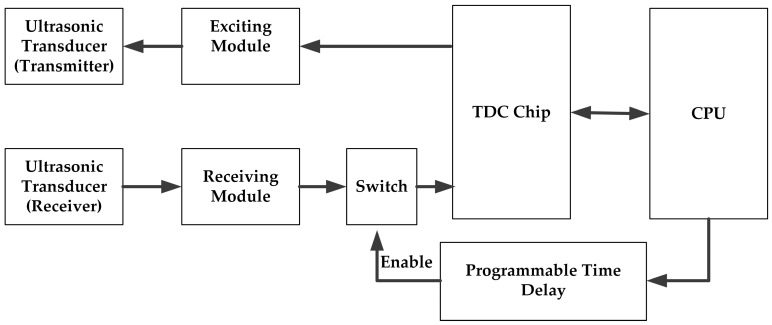
Fundamental architecture of the ultrasonic sensor. CPU: central processing unit; TDC: time-to-digital converter.

The fundamental architecture of the ultrasonic sensor for pressure measurement is shown in Figure 3. The sensor consists of a central processing unit (CPU), a Time-to-Digital Converter (TDC) chip, an exciting module and a receiving module, an ultrasonic transducer, and a switch. The TDC chip is used to generate the exciting signal for the transmitter. The exciting module can amplify the exciting signal. The ultrasonic wave received by the receiver is amplified by the receiving module, and then input into the TDC chip to measure the propagation time. The switch can be turned on or off by the CPU. Variable time delay can be set to capture the propagation time of the L_CR_ wave and the *i*th (*i* = 1, 2, …) reflected longitudinal wave. According to the sequential arrangement on the time line, the L_CR_ wave and the reflected longitudinal wave can be separated by the programmable time delay and switch. A TDC chip (TDC-GP21) produced by ACAM™ is used for precise time measurement, which has a measurement range of 3.5 ns (0 ns) to 2.5 μs with the typical resolution of 45 ps (in measurement mode 1).

### 3.2. The Experimental System

**Figure 4:**
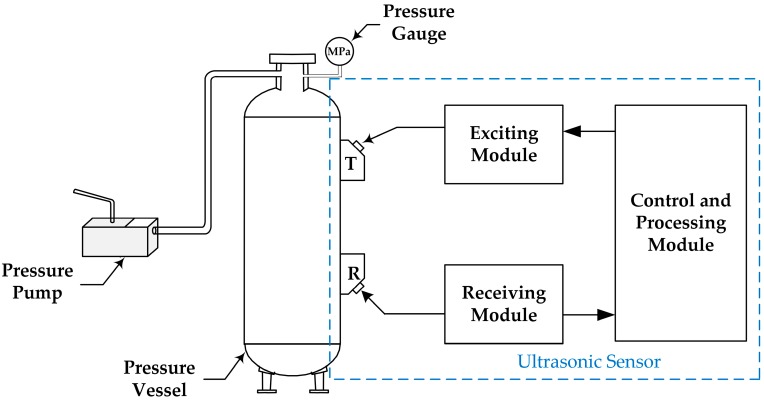
Experimental system.

We developed a prototype of the proposed ultrasonic sensor and tested it in our experimental system, as shown in Figure 4. The system consists of a pressure pump, a pressure vessel, a digital pressure gauge, and an ultrasonic sensor, which includes two ultrasonic probes: a transmitting probe (T) and a receiving probe (R), ultrasonic exciting and receiving modules, and the control and processing module.

The pressure pump (model number: SB-10, Shanghai Liyu Metal Co., Ltd., Shanghai, China) is employed to change the pressure in the pressure vessel. A digital pressure gauge (model number: NY-YBS-C, Jiangsu Nuoyi Automatic Instrument Co., Ltd., Jiangsu, China) with the full scale of 10 MPa and an error of no more than 0.02 MPa is utilized to meter the actual pressure in the pressure vessel. Table 1 shows the properties of the pressure vessel. Of the ultrasonic sensor, the ultrasonic probes have a frequency of 5 MHz, and their separation is 110 mm.

## 4. Results Analysis

In the experiments, the receiving probe receives the L_CR_ Wave and a series of reflected longitudinal waves. Waves which are detected with high SNR are considered in the construction of the measurement models. In our experiments (shown in Figure 2), the L_CR_ wave, Lre-1st wave, Lre-2nd wave, Lre-3rd wave, Lre-4th wave, Lre-5th wave, Lre-6th wave, and Lre-7th waves are qualified and therefore selected.

### 4.1. Change in Travel-Time with Temperature and Pressure

**Figure 5:**
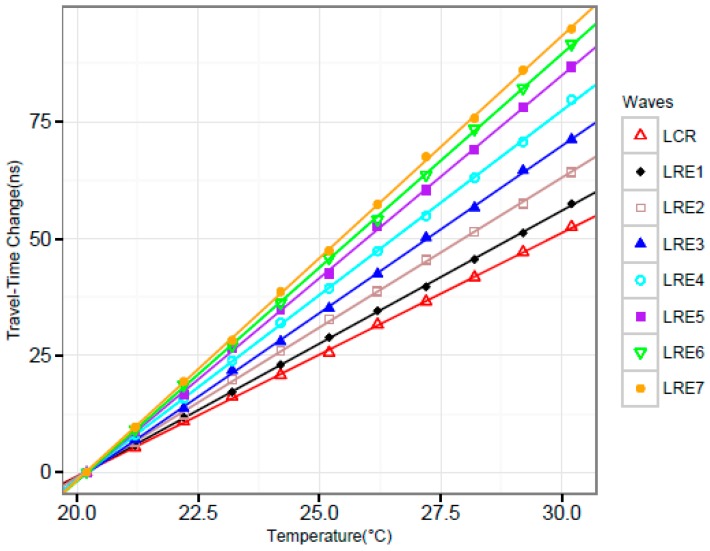
Travel-time change of multiple waves with temperature at zero pressure.

Considering the influence of temperature on the travel-time of waves, we controlled the temperature of the pressure vessel ranging from 20.2 °C to 30.2 °C with an interval of 1 °C in the experiments. The first experiment is to establish the relationship between travel-time change and temperature at zero pressure. The data collected from the experiments are shown in Figure 5. 

The lines in different colors are the fitting results using linear regression corresponding to different waves. Most of the data points are close to the corresponding line. Additionally, the R^2^ of all the regression results are above 0.98. It can be concluded that the travel-time change was linearly proportional to the temperature for the L_CR_ wave and the reflected longitudinal waves.

**Figure 6:**
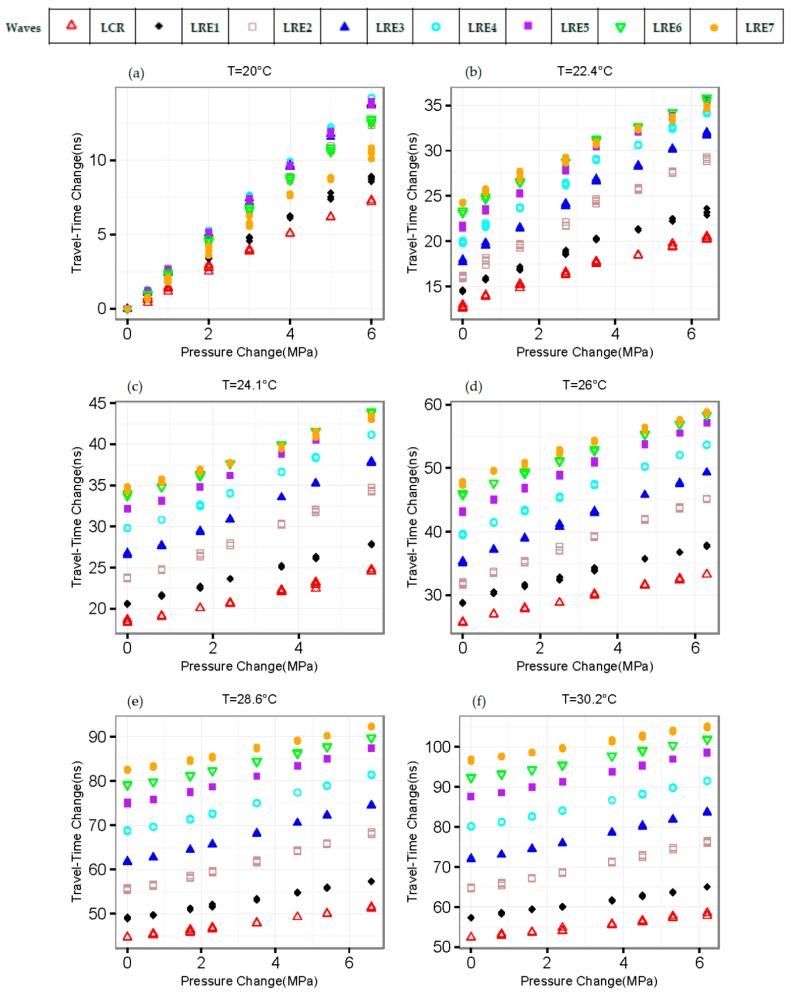
Travel-time change of multiple waves with pressure at different temperature changes. (**a**–**f**) corresponds to different temperatures.

**Figure 7:**
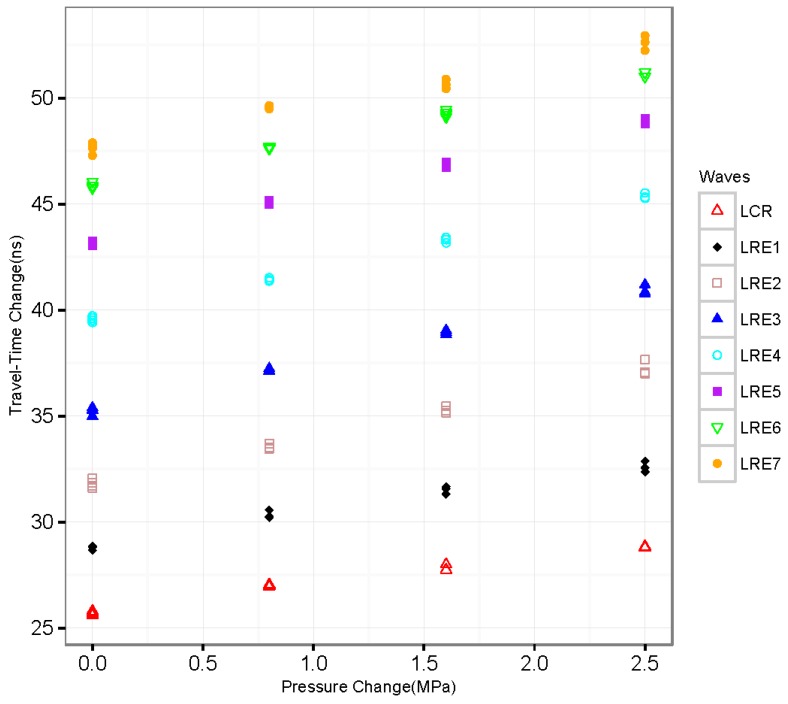
Travel-time change of multiple waves with pressure (T = 26 °C).

Once we can determine the relationship between travel-time change ($\Delta t^{\left(p,\Delta T\right)}$, p and ΔT are pressure and temperature change respectively) and temperature (T), we also need to understand the relationship between $\Delta t^{\left(p,\Delta T\right)}$ and p. Figure 6 shows the data collected from our experiments. In the past research, linear regression analysis was applied to develop the relationship between travel-time change and pressure [6]. However, the relationship between travel-time change and pressure is not perfectly linear—especially in the low-pressure zone, as shown in Figure 7. The nonlinearity might be caused by the existence of residual stress.

### 4.2. Measurement Models Based on Different Waves

Based on experimental data and the relationships we have identified, the pressure measurement models can be established [16].

The pressure measurement model based on the L_CR_ wave with temperature compensation (Model_LCR_T) can be described as Equation (5a); From Figure 6a, we can see that Lre-4th has the highest sensitivity of travel-time change with pressure. The pressure measurement model based on the Lre-4th wave with temperature compensation (Model_LRE4_T) can be described as Equation (5b); the pressure measurement model based on multiple waves (Model_Linear) can be described as Equation (5c), where coefficients $A_{1}$, $B_{1i}$, and $C_{1}$ are listed in Table 2; the pressure measurement model based on multiple waves with temperature compensation (Model_Linear_T) can be described as Equation (5d), where coefficients $A_{2}$, $B_{2i}$, $C_{2}$, and $E_{2}$ are listed in Table 3.

Considering the nonlinearity between travel-time change and pressure in the low-pressure zone, the nonlinear model (Model_Quadratic) is proposed (which can be described as Equation (5e)), where coefficients $A_{3i}$, $B_{3i}$, $C_{3}$, and $D_{3i}$ are listed in Table 4. The nonlinear model with temperature compensation (Model_Quadratic_T) can be described as Equation (5f), where coefficients $A_{4i}$, $B_{4i}$, $C_{4},\;D_{4i}$, and E4 are listed in Table 5.

(5a) $$p=0.5384\cdot \Delta t_{L_{CR}}^{\left(p,\Delta T\right)}-2.7075\Delta T+1.3420$$

(5b) $$p=0.3385\cdot \Delta t_{Lre-4^{th}}^{\left(p,\Delta T\right)}-2.5834\cdot \Delta T+0.8243$$

(5c) $$p=A_{1}\cdot \Delta t_{L_{CR}}^{\left(p,\Delta T\right)}+\sum B_{1i}\cdot \Delta t_{Lre-i^{th}}^{\left(p,\Delta T\right)}+C_{1}$$

(5d) $$p=A_{2}\cdot \Delta t_{L_{CR}}^{\left(p,\Delta T\right)}+\sum B_{2i}\cdot \Delta t_{Lre-i^{th}}^{\left(p,\Delta T\right)}+E_{2}\cdot \Delta T+C_{2}$$

(5e) $$p=A_{31}\cdot \Delta t_{L_{CR}}^{\left(p,\Delta T\right)}+A_{32}\cdot {\left(\Delta t_{L_{CR}}^{\left(p,\Delta T\right)}\right)}^{2}+\sum B_{3i}\cdot \Delta t_{Lre-i^{th}}^{\left(p,\Delta T\right)}+\sum D_{3i}\cdot {\left(\Delta t_{Lre-i^{th}}^{\left(p,\Delta T\right)}\right)}^{2}+C_{3}$$

(5f) $$p=A_{41}\cdot \Delta t_{L_{CR}}^{\left(p,\Delta T\right)}+A_{42}\cdot {\left(\Delta t_{L_{CR}}^{\left(p,\Delta T\right)}\right)}^{2}+\sum B_{4i}\cdot \Delta t_{Lre-i^{th}}^{\left(p,\Delta T\right)}+\sum D_{4i}\cdot {\left(\Delta t_{Lre-i^{th}}^{\left(p,\Delta T\right)}\right)}^{2}\;+E_{4}\cdot \Delta T+C_{4}$$

### 4.3. Experimental Results for Pressure Measurement

In Table 6, we compared the coefficient of determination (R^2^), the adjusted R^2^, and the root-mean-square error (*RMSE*) of different models.

**Figure 8:**
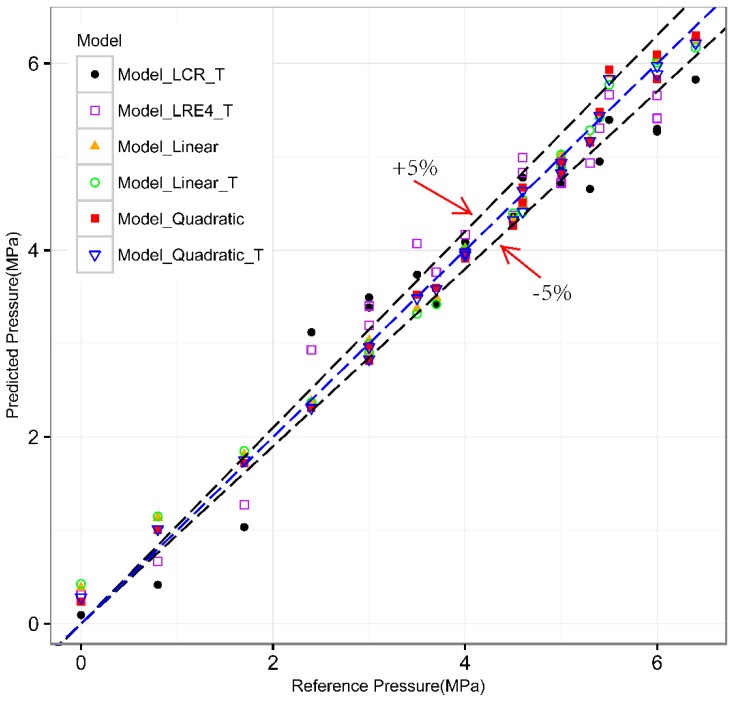
The predicted pressure vs. reference pressure.

In order to evaluate the accuracy of pressure measurement models, we analyzed the test data set from the experiment in which the temperature ranges from 20 to 30.2 °C and the pressure ranges from 0 to 6.6 MPa. Figure 8 shows the predicted pressure and the reference pressure (measured by the pressure gauge). The area between the dashed lines tagged with +5% and −5% has relative error less than 5%. The middle line indicates where the predicted pressure equals the reference pressure. From the analysis of these experiments, it is reasonable to believe that the first two models (Model_LCR_T and Model_LRE4_T) have a lower accuracy than the last four models (Model_Linear, Model_Linear_T, Model_Quadratic, and Model_Quadratic_T). And the mean relative error (MRE) (excluding data whose pressure equals zero) of the last four models is 4.3188%, 4.5328%, 3.7793%, 3.6925%, respectively. 

The results show that models based on multiple waves (Model_Linear, Model_Linear_T, Model_Quadratic, and Model_Quadratic_T) are more accurate than models based on single wave (Model_LCR_T and Model_LRE4_T). The nonlinear models with quadratic terms (Model_Quadratic and Model_Quadratic_T) work better than linear models based on multiple waves (Model_Linear, Model_Linear_T). Models without temperature compensation (Model_Linear and Model_Quadratic) can achieve same-level accuracy as models with temperature compensation (Model_Linear_T and Model_Quadratic_T). 

## 5. Conclusions

In this paper, a new mechanism of pressure measurement based on ultrasonic waves is proposed. A prototype of the ultrasonic sensor is developed and tested in a series of experiments; we can conclude that it is suitable to measure the pressure inside cylindrical pressure vessels by measuring the travel time of various longitudinal waves. In the experiments, we identified that the change in travel time of the critically refracted longitudinal wave (L_CR_ wave) and the reflected longitudinal waves vary linearly with the pressure. By applying a data fusion algorithm, the measurement models of selected waves—including L_CR_ wave and several reflected longitudinal waves—are established. Through experiments at several temperatures, we can conclude that the measurement models which take multiple waves into account will achieve higher accuracy than models using a single wave because the models of multiple waves can significantly mitigate the interference of temperature. In addition, we also found in our experiments that the model with quadratic terms would be more accurate.

To acquire the accurate travel-time change of various waves, not only a new mechanism of measurement but also a set of adequate devices are essential; for example, an analog circuit based on TDC is important in the pressure sensor.

## Figures and Tables

**Table 1:** Properties of the pressure vessel.

Component	37 Mn
Outer Radius (mm)	109.5
Inner Radius (mm)	103.8
Thickness (mm)	5.7
Height (mm)	725

**Table 2:** Coefficients of Model Linear.

**Coefficient**	$A_{1}$	$B_{11}$	$B_{12}$	$B_{13}$	$B_{14}$
Value	−0.0097	0.4370	0.1556	0.2165	1.5668
**Coefficient**	$B_{15}$	$B_{16}$	$B_{17}$	$C_{1}$	
Value	−0.6668	−1.0711	−0.1895	−0.2326	

**Table 3:** Coefficients of Model Linear_T.

**Coefficient**	$A_{2}$	$B_{21}$	$B_{22}$	$B_{23}$	$B_{24}$
Value	−0.1209	0.3394	0.1382	0.5761	1.4274
**Coefficient**	$B_{25}$	$B_{26}$	$B_{27}$	$E_{2}$	$C_{2}$
Value	−0.8771	−1.1323	−0.1212	0.2488	−0.1637

**Table 4:** Coefficients of Model Quadratic.

**Coefficient**	$A_{31}$	$A_{32}$	$B_{31}$	$B_{32}$	$B_{33}$	$B_{34}$
Value	0.3082	−0.5936	0.5960	0.4623	0.2528	0.9679
**Coefficient**	$B_{35}$	$B_{36}$	$B_{37}$	$D_{31}$	$D_{32}$	$D_{33}$
Value	−0.5936	−1.1383	−0.1933	−0.0026	−0.0041	0
**Coefficient**	$D_{34}$	$D_{35}$	$D_{36}$	$D_{37}$	$C_{3}$	
Value	0.0058	0	0	0	−0.1752	

**Table 5:** Coefficients of Model Quadratic_T.

**Coefficient**	$A_{41}$	$A_{42}$	$B_{41}$	$B_{42}$	$B_{43}$	$B_{44}$
Value	0.4347	−0.0051	0.4799	0.5627	0.6848	1.2265
**Coefficient**	$B_{45}$	$B_{46}$	$B_{47}$	$D_{41}$	$D_{42}$	$D_{43}$
Value	−1.4960	−1.1553	0	−0.0024	−0.0043	0
**Coefficient**	$D_{44}$	$D_{45}$	$D_{46}$	$D_{47}$	$E_{4}$	$C_{4}$
Value	0	0.0065	0	−0.0010	0.3221	−0.1742

**Table 6:** Comparisons between different models.

Model	R^2^	Adjusted R^2^	*RMSE*
Model_LCR_T	0.5977	0.5937	1.3229
Model_LRE4_T	0.7210	0.7183	1.1016
Model_Linear	0.9909	0.9905	0.2020
Model_Linear_T	0.9917	0.9913	0.1930
Model_Quadratic	0.9926	0.9921	0.1840
Model_Quadratic_T	0.9935	0.9931	0.1724
